# Risk Factors for Inadequate Bowel Preparation Before Colonoscopy in Patients with Ulcerative Colitis in Clinical and Endoscopic Remission: A Multicenter Retrospective Cohort Study

**DOI:** 10.3390/diagnostics16030490

**Published:** 2026-02-05

**Authors:** Davide Scalvini, Stiliano Maimaris, Elisa Stasi, Marco Valvano, Daniele Brinch, Mario Romeo, Michele Dota, Marcello Dallio, Virginia Gregorio, Chiara Sophie Sabbione, Marta Vernero, Giovanni Santacroce, Stefano Mazza, Simona Agazzi, Aurelio Mauro, Alessandro Federico, Annalisa Schiepatti, Davide Giuseppe Ribaldone, Marco Vincenzo Lenti, Gianpiero Manes, Antonio Facciorusso, Antonio Di Sabatino, Federico Biagi, Cristina Bezzio, Simone Saibeni, Andrea Anderloni

**Affiliations:** 1 Department of Internal Medicine and Medical Therapeutics, University of Pavia, 27100 Pavia, Italym.dota@universitadipavia.it (M.D.);; 2 Gastroenterology and Digestive Endoscopy Unit, Fondazione IRCCS Policlinico San Matteo, 27100 Pavia, Italy; 3 Istituti Clinici Scientifici Maugeri IRCCS, Gastroenterology Unit of Pavia Institute, 27100 Pavia, Italy; 4 Gastroenterology Unit, Department of Experimental Medicine, University of Salento, 73100 Lecce, Italy; 5 Gastroenterology Unit, Galliera Hospital, 16128 Genova, Italy; marco.valvano@galliera.it; 6 Gastroenterology and Digestive Endoscopy Unit, Ospedale Giovanni Paolo II, 97100 Ragusa, Italy; danielebrinch@gmail.com; 7 Hepatogastroenterology Division, University of Campania Luigi Vanvitelli, 80138 Napoli, Italy; mario.romeo@unicampania.it (M.R.);; 8 Endoscopy Unit, ASST Santi Paolo e Carlo, 20146 Milano, Italy; 9 Department of Medical Sciences, Gastroenterological Clinic, University of Turin, 10124 Turin, Italy; 10 First Department of Internal Medicine, Fondazione IRCCS Policlinico San Matteo, 27100 Pavia, Italy; 11 Gastroenterology Unit, Rho-Garbagnate Hospital, ASST Rhodense, 20024 Milan, Italy; 12 Department of Biomedical Sciences, Humanitas University, 20072 Pieve Emanuele, Italy

**Keywords:** bowel preparation, ulcerative colitis, colonoscopy, 1L-PEG-ASC, 2L-PEG, colorectal cancer

## Abstract

**Background/Objectives**: Adequate bowel preparation (BP) is crucial for effective colorectal cancer (CRC) surveillance in ulcerative colitis (UC). While active inflammation is known to negatively impact cleansing, data regarding predictors of BP quality specifically in UC patients with inactive disease remain limited. This study aimed to investigate risk factors for inadequate BP in UC patients in clinical/endoscopic remission and to compare the efficacy of 1L-PEG-ASC versus 2L-PEG regimens. **Methods**: A multicentric, retrospective, cohort study was conducted across eight Italian centers. Consecutive adult outpatients with UC undergoing colonoscopy between January-2021 and December-2022 who were in endoscopic and clinical remission were included. Boston Bowel Preparation Scale (BBPS) was assessed in patients undergoing 1L-PEG-ASC or 2L-PEG bowel preparation. Univariable and multivariable logistic regression analyses were performed to identify risk factors for inadequate BP and compare outcomes between PEG regimens. **Results**: A total of 379 patients were included (58% M, mean age 52.3 ± 15.4 years). The overall rate of adequate BP was 90.5%. Traditional risk factors, including demographic, clinical, and endoscopic characteristics, were not predictive of inadequate preparation in this remission cohort. Comparing regimens, 1L-PEG-ASC yielded significantly higher median total BBPS scores compared to 2L-PEG (8 [IQR 7–9] vs. 6 [IQR 6–8]; *p* < 0.001) and a higher exam completion rate (99.5% vs. 95.7%; *p* = 0.02), although the difference in adequate BP rates did not reach statistical significance (92.6% vs. 87.7%; *p* = 0.12). Multivariable analysis confirmed that 2L-PEG was independently associated with lower odds of achieving higher BBPS scores (OR 0.30; 95% CI 0.20–0.45). **Conclusions**: In UC patients with clinical and endoscopic remission, BP adequacy rates are high and comparable to the general population, suggesting that traditional IBD-related risk factors are less relevant in the absence of active inflammation. However, the 1L-PEG-ASC regimen demonstrated superior cleansing quality and exam completion rates compared to 2L-PEG. These findings support the prioritization of 1L-PEG-ASC to optimize mucosal visualization during CRC surveillance in this population.

## 1. Introduction

Ulcerative colitis (UC) is a chronic inflammatory bowel disease (IBD) characterized by continuous mucosal inflammation extending from the rectum proximally to the cecum [1]. Patients with UC require regular colonoscopy surveillance due to their increased risk of developing IBD-associated colorectal cancer and the need to monitor disease activity [2]. One of the key features for the success of colonoscopy depends on the quality of bowel preparation (BP), as inadequate cleansing can lead to a lower adenoma detection rate, higher missed lesions, and higher risk of post-colonoscopy colorectal cancer both in the general population and IBD patients [3,4,5].

Despite the obvious importance of adequate bowel preparation in IBD patients, the UC population presents unique challenges. The presence of inflammation alters bowel habits, and various disease-related factors may influence the quality of bowel preparation [6]. Furthermore, these patients undergo repeated colonoscopies throughout their lives, making the optimization of preparation strategies particularly relevant for their willingness to repeat.

While numerous studies have investigated factors affecting bowel preparation quality in the general population, specific data regarding UC patients remain limited [7,8,9]. The volume of preparation solution, timing of administration, and patient-related factors might have distinct implications in this particular group. Multiple studies demonstrated that several factors such as endoscopic disease extension, endoscopic/clinical activity, or previous surgery may interfere with the quality of BP in IBD patients [3,9,10,11].

Considering the increasing incidence of UC and the burden of repeated colonoscopies for patients and the health care system, understanding these factors is crucial for developing targeted strategies to improve colonoscopy effectiveness in CRC surveillance, where patients are generally in clinical and endoscopic remission [12]. This study aims to investigate the demographic, clinical, and endoscopic factors influencing BP quality specifically in UC patients who are in clinical and endoscopic remission. By focusing on this cohort, we sought to identify whether factors independent of active inflammation, similar to those seen in the general screening population, affect cleansing outcomes in this setting.

## 2. Materials and Methods

This is a multicentric, retrospective, cohort study performed at eight Italian centers. The study population includes consecutive patients with UC who underwent colonoscopy for disease activity monitoring or CRC screening between January 2021 and December 2022 (24 months). We included patients with clinical remission and without evidence of endoscopic activity, evaluated and classified with a partial Mayo score, Mayo endoscopic score (MES), or ulcerative colitis endoscopic index of severity (UCEIS) score for the endoscopic part [13,14,15].

### 2.1. Bowel Preparation and Colonoscopies

At each participating center, patients received standard instructions for BP, along with a list of available bowel preparations, without any specific allocation. For the purposes of this study, we included only common and extensively used BP laxatives available in Italy: Moviprep^®^ (PEG 3350 + ascorbate + electrolytes; Norgine, Harefield, UK) and Clensia^®^ (PEG 4000 + citrate + simethicone + electrolytes; Alfasigma, Milan, Italy) in the 2L-PEG group and Plenvu^®^ (PEG 3350 + ascorbate + electrolytes; Norgine, Harefield, UK) in the 1L-PEG-ASC group. See the Supplementary Materials for the extensive composition of the bowel preparations included.

Bowel preparation quality was assessed at all participating centers at the time of colonoscopy by the endoscopists performing the examination (no blinding). The Boston Bowel Preparation Scale (BBPS), which is widely recognized for its high interobserver reliability, was used to evaluate the BP quality. The BBPS represents the sum of scores assigned to the three colonic segments (right, transverse, and left), each graded on a scale from 0 to 3, resulting in a total score ranging from 0 to 9. Both segmental and total BBPS scores were recorded. Adequate bowel preparation was defined as a total BBPS score ≥ 6, with a minimum score of ≥2 in each colonic segment. All the colonoscopies were conducted using high-definition colonoscopes by endoscopists trained for IBD evaluation.

### 2.2. Inclusion and Exclusion Criteria

We included consecutive outpatients with UC aged > 16 years who underwent scheduled colonoscopy between January 2021 and December 2022 (24 months) following a split-dose BP with any of the BP laxatives included. We excluded inpatients, patients who underwent colonoscopy in an urgent or emergency setting, those with clinical activity (partial Mayo score > 2) or endoscopic activity (MES ≥ 1 or UCEIS ≥ 2), those who did not complete colonoscopy due to intolerance or high risk of perforation, and individuals who used picosulphate-based BP, other BP regimens/kit, or data on the specific BP kit used were missing. Lastly, patients with ileal or colonic surgery or with an ostomy or a PEG/J were excluded.

### 2.3. Data Collection

For the purpose of this study, the following clinical and demographic data on study participants were retrospectively collected from medical records: gender, age at colonoscopy, smoking status, previous appendicectomy, previous maximal disease extension according to Montreal classification [16,17], history of advanced therapy with biological therapy or small-molecule (as a surrogate of moderate to), and type of therapy if present. Furthermore, the following endoscopic data were collected: BP regimen and kit used, bowel preparation adequacy, BBPS, right colon cleansing, exam completion, and presence of CRC or pseudopolyps.

### 2.4. Study Outcomes

The primary outcomes of the study were to identify factors associated with inadequate bowel preparation in this UC patient cohort.

The secondary outcomes of the study were to identify differences between the group undergoing BP with 1L-PEG-ASC and the 2L-PEG group.

### 2.5. Statistical Analysis

Statistical analysis was performed using R version 4.3.1 [18] . Categorical variables were summarized as total counts and percentages and compared among groups with Fisher’s exact test. Odds ratios (ORs) with 95% confidence intervals (95% CI) were calculated. Continuous variables were summarized as mean and standard deviation (SD) or median and interquartile range (IQR) for skewed data and were compared between groups with the Mann–Whitney U test or unequal variances t-test, as appropriate. Comparisons were made both between patients with adequate and inadequate BP and between the two BP groups (1L-PEG-ASC, 2L-PEG). To investigate independent predictors of preparation quality, multivariable ordinal logistic regressions were conducted with the total BBPS score as the dependent variable. The analysis was adjusted for age, sex, and maximal disease extension. The proportional odds assumption was verified using the Brant test, and multicollinearity was assessed using Variance Inflation Factors (VIFs). Adjusted ORs and 95% CI were calculated. A post hoc sensitivity analysis, corrected for multiplicity according to Bonferroni, was conducted to compare differences in BP quality between different products within the 2L-PEG group. Two-sided *p*-values < 0.05 were considered statistically significant for all analyses.

### 2.6. Ethics

All patients gave informed consent for the collection of aggregated and anonymous data at the time of endoscopy. The study protocol conforms to the Declaration of Helsinki (6th revision, 2008) and it was approved by the Ethical Committee of Fondazione IRCCS Policlinico San Matteo, Pavia, Italy (protocol number 25375/2023). All results of the study are reported in this paper; additional data can be shared upon reasonable request to the corresponding author. The STROBE guidelines were followed for quality assurance.

## 3. Results

**Figure 1:**
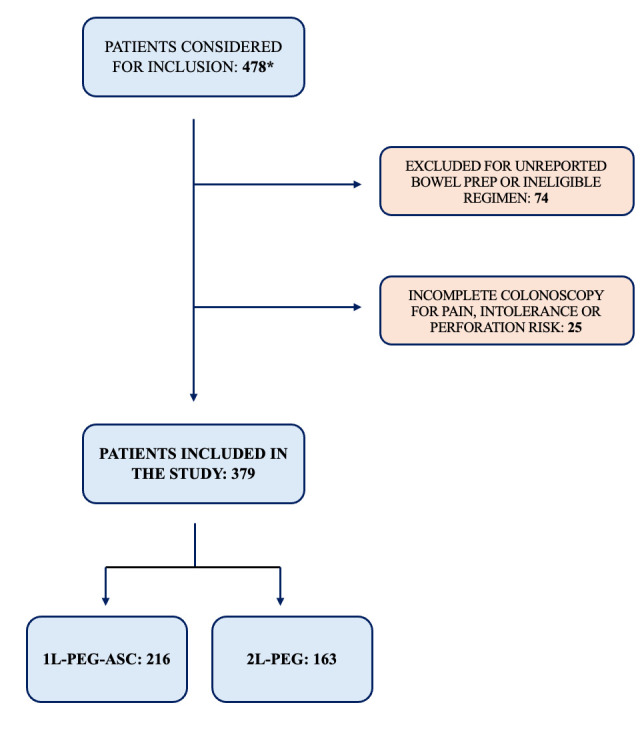
Flowchart diagram of patients included and excluded from the study. * Patients considered for inclusion were all in clinical and endoscopic remission. Patients with clinical/endoscopic activity or patients who underwent colonic resection or non-split BP were not considered, as they were ineligible for inclusion.

Overall, 379 patients were enrolled in the study (male 220, 58%) with a mean age of 52.3 ± 15.4 years. Figure 1 shows patients included and excluded from the study. In the entire cohort, the rate of adequate bowel preparation was 90.5% (343/379), while adequate cleansing of the right colon was 95.5% (362/379). Table 1 summarizes the baseline characteristics of the cohort.

The analysis of risk factors for inadequate BP, including patient demographics, smoking status, maximum disease extension, history of advanced therapy, presence of pseudopolyps, and BP volume, did not reveal any statistically significant predictors of inadequate preparation (Table 2).

**Figure 2:**
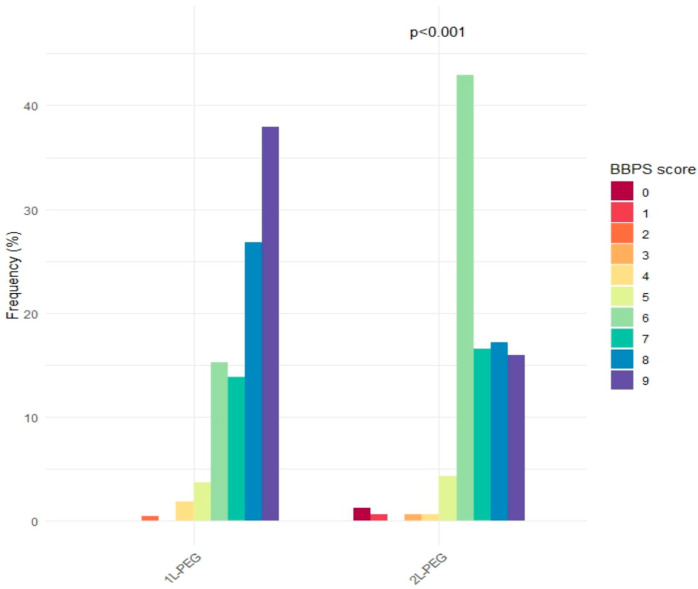
Distribution of BBPS score in 1L-PEG-ASC and 2L-PEG group.

The 1L-PEG-ASC group demonstrated a higher rate of adequate BP preparation compared to the 2L-PEG group, although this difference did not reach statistical significance (92.6% vs. 87.7%; *p* = 0.12). However, 1L-PEG-ASC yielded superior overall BP quality, with a significantly higher median total BBPS score compared to the 2L-PEG group (median 8, IQR 7–9 vs. median 6, IQR 6–8; *p* < 0.001) (Figure 2).

Multivariable ordinal logistic regression adjusted for age, sex, and previous maximal disease extension confirmed that the use of 2L-PEG was independently associated with significantly lower odds of achieving higher BBPS scores (OR 0.30; 95% CI 0.20–0.45) relative to 1L-PEG-ASC.

No significant difference was observed between the two groups regarding right colon cleansing adequacy (96.3% vs. 94.5%; *p* = 0.46). Finally, the 1L-PEG-ASC group also demonstrated a significantly higher rate of examination completion compared to the 2L-PEG group (99.5% vs. 95.7%; *p* = 0.02), further supporting the superior preparation quality associated with this BP regimen (Table 3).

Finally, a post hoc sensitivity analysis adjusted for multiplicity was conducted comparing 2L-PEG bowel preparations included in the study (Moviprep vs. Clensia), which did not reveal any significant difference in terms of adequate BP (58/64, 91% vs. 85/99, 86%; *p* = 1.00) and BBPS (median 7, IQR 6–8 vs. median 6, IQR 6–8; *p* = 0.11).

## 4. Discussion

This study investigated the presence of risk factors for inadequate bowel preparation and the effectiveness of different BP volumes in a relatively unbiased clinical context: patients with UC in clinical and endoscopic remission. This subgroup represents the target population for CRC surveillance colonoscopies and the most frequent IBD subgroup undergoing colonoscopy.

The main finding of this study is that when patients with established risk factors, such as active endoscopic disease or previous surgery, are excluded, other potential risk factors appear to be of little relevance. This underscores the dominant influence of these two predictors on BP quality in the IBD population [6,10,11]. While IBD patients are generally at higher risk for suboptimal BP, often attributed to disease extent, clinical activity, and intestinal motility alterations, our results demonstrate that UC patients in remission achieve cleansing quality comparable to, or even exceeding, that of the general population.

In this cohort, the adequate preparation rate was 90.5%, surpassing the 90% threshold recommended by the ESGE guidelines [19]. This is noteworthy as studies on the general population often fall below this target [8,20,21]. The high performance in this group may be explained by “procedural literacy”; undergoing multiple colonoscopies over a lifetime likely improves a patient’s ability to strictly adhere to instructions and select appropriate BP regimens, a benefit further amplified by clinical and endoscopic stability [22].

Unlike Kumar et al., who identified moderate-to-severe endoscopic activity and advanced therapy as risk factors for inadequate BP (with a considerable rate of 24.8% of inadequate BP in this study), our study did not find advanced therapy to be an independent risk factor [10]. We postulate that Kumar’s findings associating advanced therapy with poor preparation were likely confounded by active endoscopic disease, which was excluded in our cohort. Furthermore, although we hypothesized that pseudopolyps might serve as a marker for previous severe disease, their presence did not differ between the adequate and inadequate BP groups. Similarly, while we theorized that disease extension might impair peristalsis, our data align with other studies confirming that disease extent does not influence BP quality [11,22,23,24].

Lastly, we compared the effectiveness of different BP volumes. In the general population, 1L-PEG-ASC has already shown evidence of superiority compared to higher volumes, but there is a lack of data in IBD, specifically in UC in clinical and endoscopic remission [8,23,24,25,26,27]. While both regimens achieved satisfying adequacy rates, 1L-PEG-ASC demonstrated superior overall cleansing quality, yielding significantly higher median BBPS scores compared to the 2L-PEG group. Multivariable analysis confirmed that 1L-PEG-ASC was independently predictive of higher BBPS scores. These results are clinically vital for IBD surveillance, where high-quality mucosal visualization is essential for detecting the flat, subtle dysplastic lesions characteristic of ulcerative colitis. Unlike sporadic adenomas, dysplasia in UC is often non-polypoid and subtle. Consequently, adequate (BBPS ≥ 6) preparation may not be the optimal target in this population, and excellent preparation is likely to be preferable. Therefore, the significantly higher total BBPS scores observed with 1L-PEG-ASC argue for its preferential use to maximize dysplasia detection rates. Moreover, the higher exam completion rate with 1L-PEG-ASC compared to 2L-PEG leads to fewer repeat examinations, also leading to cost savings and a lower carbon footprint.

This study has several limitations that warrant consideration. Firstly, its retrospective design may have introduced inherent selection bias and restricted control over data collection, although the inclusion of eight centers helps mitigate this. Secondly, we lacked data on concomitant comorbidities and medications, such as antidepressants, antidiabetics, or opioids, which are known to potentially influence gastrointestinal motility and bowel preparation quality. The relatively small number of patients with inadequate BP in our sample may have limited the statistical power of our analysis in capturing potentially significant risk factors and limited the number of variables we could include in the multivariable analysis. Finally, disease duration was not recorded; however, the existing literature suggests that disease duration does not significantly impact preparation quality in IBD patients [28].

## 5. Conclusions

In conclusion, this large multicenter cohort shows that among patients with UC who are in both clinical and endoscopic remission, BP adequacy meets ESGE-recommended thresholds. In this stable population, traditional IBD-related risk factors do not appear to adversely affect bowel cleansing and, given the frequent colonoscopic surveillance these patients undergo, they may not require prioritization for enhanced pre-colonoscopy instruction. Notably, the 1L-PEG-ASC regimen served as an independent protective factor against inadequate preparation and yielded significantly higher BBPS scores. These findings suggest that 1L-PEG-ASC is a highly effective option for optimizing mucosal visualization during CRC surveillance. Despite the study’s limitations, these results provide valuable real-world evidence for optimizing preparation protocols in the IBD population.

## Figures and Tables

**Table 1:** Baseline characteristics of the cohort.

Characteristics		Overall (379 Patients)
**Age (mean (SD))**		52.3 (15.4)
**BBPS total (median [IQR])**		8.00 [6.00, 9.00]
**Age (%)**		
	<40	81 (21.3%)
	40–59	164 (43.3%)
	>60	134 (35.4%)
**Gender (%)**	F	159 (42.0%)
	M	220 (58.0%)
**Active smoking (%)**	No (0)	336 (88.7%)
	Yes (1)	43 (11.3%)
**Appendicectomy (%)**	No (0)	352 (92.9%)
	Yes (1)	27 (7.1%)
**Previous maximal disease extension (%)**	E1	58 (15.5%)
	E2	153 (40.9%)
	E3	163 (43.6%)
**History of advanced therapy (%)**	No (0)	214 (56.5%)
	Yes (1)	165 (43.5%)
**Bowel preparation volume (%)**	1L-PEG	216 (57.0%)
	2L-PEG	163 (43.0%)
**Bowel preparation kit (%)**	Plenvu	216 (57%)
	Clensia	99 (26%)
	Moviprep	64 (17%)
**Adequate bowel preparation (%)**	No (0)	36 (9.5%)
	Yes (1)	343 (90.5%)
**Adequate right colon cleansing (%)**	No (0)	17 (4.5%)
	Yes (1)	362 (95.5%)
**Exam completed (%)**	No (0)	8 (2.1%)
	Yes (1)	371 (97.9%)
**Cancer detection (%)**	No (0)	377 (99.5%)
	Yes (1)	2 (0.5%)
**Pseudopolyps (%)**	No (0)	337 (88.9%)
	Yes (1)	42 (11.1%)

**Table 2:** Risk factors for inadequate bowel preparation.

Characteristics	Level	Overall (*n* = 379)	Inadequate (*n* = 36)	Adequate (*n* = 343)	*p*-Value
**Age (mean (SD))**		52.31 (15.37)	52.4 (14.4)	52.3 (15.5)	0.957
**Age_cat (%)**	<40	81 (21.3)	6 (16.7)	75 (21.8)	0.809
	40–59	164 (43.3)	17 (47.2)	147 (42.9)	
	>60	134 (35.4)	13 (36.1)	121 (35.3)	
**Gender (%)**	F	159 (42.0)	11 (30.6)	148 (43.1)	0.159
	M	220 (58.0)	25 (69.4)	195 (56.9)	
**Active smoking (%)**	0 (No)	336 (88.7)	29 (80.6)	307 (89.5)	0.160
	1 (Yes)	43 (11.3)	7 (19.4)	36 (10.5)	
**Appendicectomy (%)**	0 (No)	352 (92.9)	33 (91.7)	319 (93.0)	0.732
	1 (Yes)	27 (7.1)	3 (8.3)	24 (7.0)	
**Maximal disease extension (%)**	E1	58 (15.5)	6 (16.7)	52 (15.4)	0.627
	E2	153 (40.9)	17 (47.2)	136 (40.2)	
	E3	163 (43.6)	13 (36.1)	150 (44.4)	
**Advanced therapy (%)**	0 (No)	214 (56.5)	18 (50.0)	196 (57.1)	0.481
	1 (Yes)	165 (43.5)	18 (50.0)	147 (42.9)	
**BP volume (%)**	1L-PEG	216 (57.0)	16 (44.4)	200 (58.3)	0.115
	2L-PEG	163 (43.0)	20 (55.6)	143 (41.7)	
**Exam completed (%)**	0 (No)	8 (2.1)	8 (22.2)	0 (0.0)	<0.001
	1 (Yes)	371 (97.9)	28 (77.8)	343 (100.0)	
**Cancer (%)**	0 (No)	377 (99.5)	36 (100.0)	341 (99.4)	1.000
	1 (Yes)	2 (0.5)	0 (0.0)	2 (0.6)	
**Pseudopolyps (%)**	0 (No)	337 (88.9)	32 (88.9)	305 (88.9)	1.000
	1 (Yes)	42 (11.1)	4 (11.1)	38 (11.1)	

**Table 3:** Comparison between 1L-PEG-ASC and 2L-PEG in outcomes.

Characteristics	Level	Overall (*n* = 379)	1L-PEG (*n* = 216)	2L-PEG (*n* = 163)	*p*-Value
**Age (mean (SD))**		52.31 (15.37)	53.28 (15.16)	51.02 (15.60)	0.157
**BBPS TOTAL (median [IQR])**		8.00 [6.00–9.00]	8.00 [7.00–9.00]	6.00 [6.00–8.00]	<0.001
**Age_cat (%)**	<40	81 (21.3)	44 (20.3)	37 (22.7)	0.475
	40–59	164 (43.3)	90 (41.7)	74 (45.4)	
	>60	134 (35.4)	82 (38.0)	52 (31.9)	
**Gender (%)**	F	159 (42.0)	95 (44.0)	64 (39.3)	0.401
	M	220 (58.0)	121 (56.0)	99 (60.7)	
**Active smoking (%)**	0	336 (88.7)	196 (90.7)	140 (85.9)	0.145
	1	43 (11.3)	20 (9.3)	23 (14.1)	
**Appendicectomy (%)**	0	352 (92.9)	206 (95.4)	146 (89.6)	0.042
	1	27 (7.1)	10 (4.6)	17 (10.4)	
**Maximal disease extension (%)**	E1	58 (15.5)	22 (10.4)	36 (22.1)	0.002
	E2	153 (40.9)	84 (39.8)	69 (42.3)	
	E3	163 (43.6)	105 (49.8)	58 (35.6)	
**Advanced therapy (%)**	0	214 (56.5)	119 (55.1)	95 (58.3)	0.601
	1	165 (43.5)	97 (44.9)	68 (41.7)	
**BP volume (%)**	1L-PEG	216 (57.0)	216 (100.0)	0 (0.0)	<0.001
	2L-PEG	163 (43.0)	0 (0.0)	163 (100.0)	
**Adequate BP (%)**	0	36 (9.5)	16 (7.4)	20 (12.3)	0.115
	1	343 (90.5)	200 (92.6)	143 (87.7)	
**Right colon cleansing (%)**	0	17 (4.5)	8 (3.7)	9 (5.5)	0.457
	1	362 (95.5)	208 (96.3)	154 (94.5)	
**Exam completed (%)**	0	8 (2.1)	1 (0.5)	7 (4.3)	0.023
	1	371 (97.9)	215 (99.5)	156 (95.7)	
**Cancer (%)**	0	377 (99.5)	215 (99.5)	162 (99.4)	1.000
	1	2 (0.5)	1 (0.5)	1 (0.6)	
**Pseudopolyps (%)**	0	337 (88.9)	187 (86.6)	150 (92.0)	0.100
	1	42 (11.1)	29 (13.4)	13 (8.0)	

## Data Availability

The original contributions presented in the study are included in the article/Supplementary Material, further inquiries can be directed to the corresponding author.

## Supplementary Materials

The following supporting information can be downloaded at: https://www.mdpi.com/article/10.3390/diagnostics16030490/s1.

## References

[1] Rubin D.T., Ananthakrishnan A.N., Siegel C.A., Barnes E.L., Long M.D. (2025). ACG Clinical Guideline Update: Ulcerative Colitis in Adults. Am. J. Gastroenterol..

[2] Laine L., Kaltenbach T., Barkun A., McQuaid K.R., Subramanian V., Soetikno R. (2015). SCENIC Guideline Development Panel. SCENIC international consensus statement on surveillance and management of dysplasia in inflammatory bowel disease. Gastroenterology.

[3] De Cristofaro E., Marafini I., Mancone R., Fiorillo M., Franchin M., Mattogno A., Neri B., Zorzi F., Blanco G.D.V., Biancone L. (2025). Preventable Predictive Factors of Post-colonoscopy Colorectal Cancer in Inflammatory Bowel Disease. J. Crohns Colitis.

[4] Scalvini D., Agazzi S., Maimaris S., Rovedatti L., Brinch D., Cappellini A., Ciccioli C., Puricelli M., Bartolotta E., Alfieri D. (2025). Strategies to Enhance the Adenoma Detection Rate (ADR) and the Serrated Polyp Detection Rate (SPDR) in Colonoscopy: A Comprehensive Review. Gastroenterol. Insights.

[5] Zessner-Spitzenberg J., Waldmann E., Rockenbauer L.M., Klinger A., Klenske E., Penz D., Demschik A., Majcher B., Trauner M., Ferlitsch M. (2024). Impact of Bowel Preparation Quality on Colonoscopy Findings and Colorectal Cancer Deaths in a Nation-Wide Colorectal Cancer Screening Program. Am. J. Gastroenterol..

[6] Gravina A.G., Pellegrino R., Romeo M., Palladino G., Cipullo M., Iadanza G., Olivieri S., Zagaria G., De Gennaro N., Santonastaso A. (2023). Quality of bowel preparation in patients with inflammatory bowel disease undergoing colonoscopy: What factors to consider? *World*. J. Gastrointest. Endosc..

[7] Hassan C., Fuccio L., Bruno M., Pagano N., Spada C., Carrara S., Giordanino C., Rondonotti E., Curcio G., Dulbecco P. (2012). A predictive model identifies patients most likely to have inadequate bowel preparation for colonoscopy. Clin. Gastroenterol. Hepatol..

[8] Scalvini D., Lenti M.V., Maimaris S., Lusetti F., Alimenti E., Fazzino E., Mauro A., Mazza S., Agazzi S., Strada E. (2024). Superior bowel preparation quality for colonoscopy with 1L-PEG compared to 2L-PEG and picosulphate: Data from a large real-world retrospective outpatient cohort. Dig. Liver Dis..

[9] Beran A., Aboursheid T., Ali A.H., Albunni H., Mohamed M.F., Vargas A., Hadaki N., Alsakarneh S., Rex D.K., Guardiola J.J. (2024). Risk Factors for Inadequate Bowel Preparation in Colonoscopy: A Comprehensive Systematic Review and Meta-Analysis. Am. J. Gastroenterol..

[10] Kumar A., Shenoy V., Buckley M.C., Durbin L., Mackey J., Mone A., Swaminath A. (2022). Endoscopic Disease Activity and Biologic Therapy Are Independent Predictors of Suboptimal Bowel Preparation in Patients with Inflammatory Bowel Disease Undergoing Colonoscopy. Dig. Dis. Sci..

[11] Scalvini D., Bezzio C., Maimaris S., Lenti M.V., Francesca L., Cappellini A., Cicalini C., Dota M., Muscia R., Brinch D. (2025). A multicenter study on bowel preparation in inflammatory bowel disease patients: Comparison between 1L-PEG-ASC and 2L-PEG regimens in an outpatient setting. Eur. J. Gastroenterol. Hepatol..

[12] Kaplan G.G. (2025). The global burden of inflammatory bowel disease: From 2025 to 2045. Nat. Rev. Gastroenterol. Hepatol..

[13] Schroeder K.W., Tremaine W.J., Ilstrup D.M. (1987). Coated oral 5-aminosalicylic acid therapy for mildly to moderately active ulcerative colitis. A randomized study. N. Engl. J. Med..

[14] Travis S.P., Schnell D., Krzeski P., Abreu M.T., Altman D.G., Colombel J., Feagan B.G., Hanauer S.B., Lichtenstein G.R., Marteau P.R. (2013). Reliability and initial validation of the ulcerative colitis endoscopic index of severity. Gastroenterology.

[15] Lewis J.D., Chuai S., Nessel L., Lichtenstein G.R., Aberra F.N., Ellenberg J.H. (2008). Use of the noninvasive components of the Mayo score to assess clinical response in ulcerative colitis. Inflamm. Bowel Dis..

[16] Calderwood A.H., Schroy P.C., Lieberman D.A., Logan J.R., Zurfluh M., Jacobson B.C. (2014). Boston Bowel Preparation Scale scores provide a standardized definition of adequate for describing bowel cleanliness. Gastrointest. Endosc..

[17] Satsangi J., Silverberg M.S., Vermeire S., Colombel J.F. (2006). The Montreal classification of inflammatory bowel disease: Controversies, consensus, and implications. Gut.

[18] R Core Team R: A Language and Environment for Statistical Computing. R Foundation for Statistical Computing.

[19] Dekker E., Nass K.J., Iacucci M., Murino A., Sabino J., Bugajski M., Carretero C., Cortas G., Despott E.J., East J.E. (2022). Performance measures for colonoscopy in inflammatory bowel disease patients: European Society of Gastrointestinal Endoscopy (ESGE) Quality Improvement Initiative. Endoscopy.

[20] Occhipinti V., Soriani P., Vavassori S., Annunziata M.L., Bagolini F., Cavallaro F., Lagoussis P., Milani V., Rondonotti E., Spina L. (2023). Risk factors for inadequate bowel preparation in patients using high- and low-volume cleansing products. Eur. J. Gastroenterol. Hepatol..

[21] López-Jamar J.M.E., Gorjão R., Cotter J., García V.L.-Z., Sánchez M.A.P., Martínez D.C., Sábado F., Arellano E.P., Rodríguez B.J.G., Cano A.L. (2023). Bowel cleansing effectiveness and safety of 1L PEG + Asc in the real-world setting: Observational, retrospective, multicenter study of over 13000 patients. Endosc. Int. Open..

[22] Maida M., Morreale G.C., Sferrazza S., Sinagra E., Scalisi G., Vitello A., Vettori G., Rossi F., Catarella D., Di Bartolo C. (2021). Effectiveness and safety of 1L PEG-ASC preparation for colonoscopy in patients with inflammatory bowel diseases. Dig. Liver Dis..

[23] Manes G., Fontana P., de Nucci G., Radaelli F., Hassan C., Ardizzone S. (2015). Colon Cleansing for Colonoscopy in Patients with Ulcerative Colitis: Efficacy and Acceptability of a 2-L PEG Plus Bisacodyl Versus 4-L PEG. Inflamm. Bowel Dis..

[24] Repici A., Spada C., Cannizzaro R., Traina M., Maselli R., Maiero S., Galtieri A., Guarnieri G., Di Leo M., Lorenzetti R. (2021). Novel 1-L polyethylene glycol + ascorbate versus high-volume polyethylene glycol regimen for colonoscopy cleansing: A multicenter, randomized, phase IV study. Gastrointest. Endosc..

[25] Bisschops R., Manning J., Clayton L.B., Ng Kwet Shing R., Álvarez-González M. (2019). MORA Study Group. Colon cleansing efficacy and safety with 1 L NER1006 versus 2 L polyethylene glycol + ascorbate: A randomized phase 3 trial. Endoscopy.

[26] Frazzoni L., Spada C., Radaelli F., Mussetto A., Laterza L., La Marca M., Piccirelli S., Cortellini F., Rondonotti E., Paci V. (2020). 1L- vs. 4L-Polyethylene glycol for bowel preparation before colonoscopy among inpatients: A propensity score-matching analysis. Dig. Liver Dis..

[27] Hong S.N., Lee C.K., Im J.P., Choi C.H., Byeon J.S., Cho Y.S., Jung S.A., Kim T.I., Jeen Y.T. (2022). Efficacy and safety of split-dose bowel preparation with 1 L polyethylene glycol and ascorbate compared with 2 L polyethylene glycol and ascorbate in a Korean population: A phase, I.V.; multicenter, randomized, endoscopist-blinded study. Gastrointest. Endosc..

[28] Negreanu L., Voiosu T., State M., Mateescu R.B. (2020). Quality of colonoscopy preparation in patients with inflammatory bowel disease: Retrospective analysis of 348 colonoscopies. J. Int. Med. Res..

