# Supplementary material for: Risk Factors for Inadequate Bowel Preparation Before Colonoscopy in Patients with Ulcerative Colitis in Clinical and Endoscopic Remission: A Multicenter Retrospective Cohort Study
Source: Diagnostics (Basel). 2026 Feb 5;16(3):490. doi: 10.3390/diagnostics16030490 (PMC12896592; doi:10.3390/diagnostics16030490)
Supplement: Supplementary file 1 [file diagnostics-16-00490-s001.zip › diagnostics-4108330-supplementary.pdf]

Supplementary Materials

Plenvu®

Dose 1. Sachet contains the following active substances: Macrogol 3350 100 g, Sodium sulfate 9 g, Sodium chloride 2 g, Potassium chloride 1 g. The concentration of electrolyte ions when the first dose is made up to 500 mL of solution is as follows: Sodium 160.9 mmol/500 mL, Sulfate 63.4 mmol/500 mL, Chloride 47.6 mmol/500 mL, Potassium 13.3 mmol/500 mL. Dose 1 also contains 0.79 g of sucralose (E955).

Dose 2 (Sachets A and B) contains the following active substances:

Sachet A: Macrogol 3350 40 g, Sodium chloride 3.2 g, Potassium chloride 1.2 g

Sachet B: Sodium ascorbate 48.11 g, Ascorbic acid 7.54 g.

The concentration of electrolyte ions when the first dose is made up to 500 mL of solution is as follows: Sodium 297,6 mmol/500 ml, Ascorbate 285,7 mmol/500 ml, Chloride 70,9 mmol/500 ml, Potassium 16,1 mmol/500 ml. Dose 1 also contains 0,88 g of aspartame (E951).

Moviprep®

It consists of 4 separate pouches (2 of pouch A and 2 of pouch B). Each pouch A contains 100 g of PEG 3350, Sodium sulfate 7.5 g, Sodium chloride 2.69 g and Potassium chloride 1.015 g. Each pouch B contains Ascorbic acid 4.7 g and Sodium Ascorbate 5.9 g.

1 pouch A and 1 pouch B should be dissolved together in 1 liter of lukewarm water. When reconstituted to 1 liter volume with water, the solution contains PEG-3350 29.6 mmol/L, sodium 181.6 mmol/L (of which not more than 56.2 mmol is ascorbate), sulfate 52.8 mmol/L, chloride 59.8 mmol/L, potassium 14.2 mmol/L, and ascorbate 29.8 mmol/L.

Clensia®

Clensia is available as a powder in 2 separate sachets (A-large and B-small) to be dissolved together in

water and administered as an oral solution.

Sachet A (large) contains the following active substances: Macrogol 4000 52.5 g, Sodium sulphate anhydrous 3.750 g, Simeticone 0.080 g

Sachet B (small) contains the following active substances: Sodium citrate 1.863 g, Citric acid anhydrous 0.813 g, Sodium chloride 0.730 g, Potassium chloride 0.370 g

The concentration of electrolyte ions when 2 sachets A and 2 sachets B are dissolved in 1 litre of water is as follows: Sodium 168.6 mmol/l, Sulphate 52.8 mmol/l, Chloride 34.9 mmol/l, Potassium 11.2 mmol/l, Citrate 21.1 mmol/l. Excipient with known effect: sachet B contains 0.130 g of acesulfame potassium.

A single treatment for bowel cleansing in adults consists of 4 sachets A and 4 sachets B dissolved in 2

litres of water taken orally.
